# CD44, TGM2 and EpCAM as novel plasma markers in endometrial cancer diagnosis

**DOI:** 10.1186/s12885-019-5556-x

**Published:** 2019-04-29

**Authors:** Anna Torres, Małgorzata Pac-Sosińska, Krzysztof Wiktor, Tomasz Paszkowski, Ryszard Maciejewski, Kamil Torres

**Affiliations:** 1 Laboratory of Biostructure, Chair of Human Anatomy, Medical University of Lublin, Lublin, Poland; 2 III Chair and Department of Gynaecology, Medical University of Lublin, Lublin, Poland; 3 Pediatric and Adolescent Gynecology Unit, University Children’s Hospital, Medical University of Lublin, Lublin, Poland; 4 Laboratory of Diagnostic Procedures, Medical University of Lublin, Lublin, Poland; 5 Collegium Anatomicum, Jaczewskiego 4, 20-090 Lublin, Poland

**Keywords:** Endometrial cancer, CD44, EpCAM, TGM2, Novel plasma markers, Endometriosis

## Abstract

**Background:**

Endometrial cancer (EC) is the most common malignancy of the female reproductive tract. Despite years of research, the accurate screening strategy is still not available in this disease and it is usually diagnosed only after the clinical signs are present. The recent technological advances in analytical methodologies enabled detection of multiple molecules in one, small sample of biological materials. Such approach was undertaken in the presented study.

**Methods:**

Concentrations of aldehyde dehydrogenase 1 family, member A1 (ALDH1A1), carbonic anhydrase IX (CA9), CD44, epithelial cell adhesion molecule (EpCAM), hepsin, kallikrein-6, mesothelin, midkine, neural cell adhesion molecule L1 (L1CAM), and transglutaminase 2 (TGM2) were measured using MAGPIX®System in plasma samples of 45 EC, 20 healthy controls and 11 patients with endometriosis.

**Results:**

Significantly increased concentration in EC as compared to healthy controls were found in case of CD44 (*p* <  0.001), EpCAM (*p* = 0.033) and TGM2 (*p* <  0.001). EpCAM and mesothelin concentrations differed based on FIGO stages. Regression analysis revealed marker panels with high accuracy in detection of EC. The highest AUC 0.937 was attributed to the 3-marker panel of CD44/TGM2/EpCAM (84% sensitivity, 100% specificity), FIGO IA samples were discriminated from more advanced stages of EC with the mesothelin/grade 1 model featuring AUC of 0.911 (95.24% sensitivity, 78.26% specificity).

**Conclusions:**

Novel plasma biomarkers presenting good accuracy in diagnosing EC were found with TGM2 reported for the first time as plasma marker. It was also revealed that endometriosis may share similarities in the pattern of markers alterations characteristic for EC.

**Electronic supplementary material:**

The online version of this article (10.1186/s12885-019-5556-x) contains supplementary material, which is available to authorized users.

## Background

Multiple serum and plasma circulating molecules have been suggested as possible biomarkers in endometrial cancer (EC) detection [1]. However, none of those proposed molecules became a recommended marker for the routine EC screening or diagnosis. Several factors justify research efforts aiming to find the screening and early detection strategy in this malignancy, and include high and consistently rising incidence of EC, lack of improvement in survival rates and the fact that EC is the most common malignancy of the female reproductive tract [2, 3].

In previous years the search for possible markers in cancer detection was based on studying single analytes in one sample. That strategy was laborious, expensive and required relatively large sample volumes. The recent technological advances in analytical methodologies enabled detection of multiple molecules in one, small sample of biological material, and offered the opportunity of developing biomarker panels, which could increase accuracy in detection of certain diseases including endometrial cancer [4]. With regard to aforementioned advantages such approach was undertaken in the presented study. The method used for the study had the capability of adding multiple conjugated beads to each sample resulting in the reception of multiple results from each sample, which saved the sample material, reduced time, labor and costs over traditional methods [4]. Based on the published literature the panel of ten potential markers was chosen to be simultaneously studied in EC samples. The panel included: aldehyde dehydrogenase 1 family, member A1 (ALDH1A1), carbonic anhydrase IX (CA9), CD44, epithelial cell adhesion molecule (EpCAM), hepsin, kallikrein-6, mesothelin, midkine, neural cell adhesion molecule L1 (L1CAM), and transglutaminase 2 (TGM2). The more detailed description of the markers and the rationale for their selection was presented in the Table 1 [5–25].

**Table 1:** Characteristics of selected biomarkers

Marker	Function	Cancers	References
ALDH1A1	stem-like cells biomarker involved in dehydrogenation of aldehydes to their corresponding carboxylic acids; plays role in differentiation, proliferation and motility	ovarian, breast, head and neck lung colorectal endometrial (ICH study)	Rahadiani et al. [5]
CA9	transmembrane HIF- 1훼-dependent glycoprotein responsible for the regulation of pH in the tumor microenvironment	colorectal, gastric, endometrial	Hynninen P et al. [6], Sadlecki et al. [7]
CD44	hyaluronic acid receptor, responsible for cell adhesion; affects carcinogenesis through cell migration and metastasis initiation; cancer stem cell marker	breast, pancreatic, gastric, hepatocellular, bladder, biliary, vulvar, endometrial (ICH study)	Wojciechowski et al. [8], Elbasateeny et al. [9], Yan et al. [10]
EpCAM	calcium-independent homophilic cell adhesion molecule of 39–42 kDa, frequently and highly expressed on carcinomas, tumor-initiating cells, selected tissue progenitors, and embryonic and adult stem cells	ovarian, breast, uterine serous cancer	El-Sahwi et al. [11], Hsu et al. [12]
Hepsin	a type II transmembrane serine protease originally identified in the human liver as a cDNA clone; Hepsin mRNA is highly expressed in normal liver tissues, while it is poorly expressed in other tissues, including normal kidney, pancreas, lung, thyroid, pituitary gland and testis	prostate, renal, ovarian, endometrial	El-Rebey et al. [13] Matsuo et al. [14]
Kallikrein 6	serine protease with roles in diverse cellular activities, including blood coagulation, wound healing, digestion, and immune responses as well as tumor invasion and metastasis	ovarian, uterine serous cancer	Santin et al. [15] Dorn et al. [16]
L1CAM	a neuronal cell adhesion transmembrane protein with a strong implication in cell migration, adhesion, neurite outgrowth, myelination and neuronal differentiation; plays a role in migration, invasion, growth metastasis and chemoresistance.	ovarian, breast, gastric, melanoma, endometrial, esophageal, colorectal, pancreatic, prostate, neuroblastoma	Bosse et al. [17] Pasanen et al. [18] Zeimet et al. [19] Tangen et al. [20]
Mesothelin	a cell-surface glycoprotein with normal expression limited to mesothelial cells lining the pleura, peritoneum; the biologic role that MSLN plays in these cells remains unclear; in cancer it increases cellular resistance to anoikis, upregulates matrix metalloproteinases important in cellular invasion and metastasis, and induces secretion of autocrine growth factors	malignant mesothelioma, pancreatic, ovarian, lung, endometrial cancer, biliary gastric, pediatric acute myeloid leukemia	Dainty et al. [21] Obulhasim et al. [22]
Midkine	a secreted, heparin-binding growth factor; a 13- kDa protein rich in basic amino acids and cysteine; is highly expressed during embryo- genesis; in the adult, the highest transcript levels are in the intestine with low levels in the cerebellum, thyroid, kidney, bladder, lung alveoli, colon, stomach, and spleen; has a role in oncogenic transformation of fibroblasts, antiapoptotic activity, and angiogenic activity	breast, lung, esophagus, colon, ovary, urinary bladder, and prostate, glioblastoma, neuroblastoma, Wilms’ tumor	Tanabe et al. [23]
TGM2	predominantly a cytosolic protein, also present in the nucleus, plasma membrane and in the extracellular environment; a marker of cancer development and cancer stem cell-survival	ovarian, breast, pancreatic, prostate, glioma, melanoma, lung, colon, leukemia	Li et al. [24] Eckert et al. [25]

The authors of the study aimed to determine markers discriminating: 1) EC and the pooled control and endometriosis samples; 2) EC and the healthy controls; 3) different FIGO stages of EC.

## Methods

### Patients and blood samples

Seventy-six females, patients of the III Department of Gynecology, Medical University of Lublin, were included in the study (EC = 45, endometriosis = 11, healthy controls = 20). Patients in the control group did not have history of malignancy, reproductive tract pathology or symptoms of endometriosis. None of the included patients had a history of any serious illness e.g. diabetes, autoimmunological, liver or kidney disease. Patients in EC and endometriosis groups did not have history of other malignancies or were submitted to neo-adjuvant therapy. The EC patients underwent total hysterectomy with bilateral salpingoophorectomy due to EC diagnosed with endometrial biopsy prior to operation. All cases were endometrioid endometrial carcinomas. After the operations patients were submitted to radiotherapy and/or chemotherapy according to International Federation of Gynecology and Obstetrics (FIGO) guidelines. Clinical stage of the disease was determined according to 2010 FIGO classification. Median age of the control samples was 51 (95% CI 46.53–56.81) and significantly differed from all EC patients (*Median* = 58, 95% CI 54.83–61.35; *p* = 0.011), but not from FIGO IA patients. There were no significant differences in patient age between subgroups distinguished based on FIGO stage, histological grade and myometrial invasion.

There were 24 FIGO IA, 12 FIGO IB and 9 FIGO III samples. Distribution of cases based on histological grades was: G1–23, G2–18, G3–4, and based on myometrial invasion was: < 0.5 thickness-26, ≥0.5 thickness-19 (Table 2, Additional file 1). According to revised American Society for Reproductive Medicine scoring system all endometriosis cases were classified as stage IV.

**Table 2:** Summary of clinicopathological characteristics of the endometrial cancer group

	FIGO stage								Grade			Invasion	
	IA	IB	II	IIIA	IIIB	IIIC1	IIIC2	IV	1	2	3	< 0.5	> = 0.5
EC (n)	24	12	0	0	3	6	0	0	23	18	4	26	19

Fasting venous blood was collected one day before the surgery from the antecubital vein (5 ml, EDTA) and was stored for maximum one hour in 4 °C until it was further processed. Blood was centrifuged for 15 min (1800 g, 19 °C) and plasma was collected. Plasma samples were aliquoted to 300 μL and were stored in − 80 °C until further analyses.

### MAGPIX®system multianalyte profiling of markers

Methodology used in the study was based on the Luminex® xMAP® technology capable of performing immunoassays on the surface of fluorescent-coded magnetic beads coated with specific capture antibodies. After an analyte from a test sample is captured by the bead, a biotinylated detection antibody is introduced. The reaction mixture is then incubated with Streptavidin-PE conjugate, the reporter molecule, to complete the reaction on the surface of each microsphere. Each individual microsphere is identified and the result of its bioassay is quantified based on fluorescent reporter signals. The study was performed with commercially available MILLIPLEX® MAP Human Circulating Cancer Biomarker Magnetic Bead Panel 4 (Merck KGaA, Darmstadt, Germany). The analytes were simultaneously detected in 25 μL samples according to manufacturer’s protocol using MAGPIX®System (Luminex Corporation, USA) and mean fluorescence intensities were calculated with the xPonent 4.2 software, using five-parameter logistic curve fitting to derive the analyte concentrations in each sample.

### Statistical analysis

Mean or median with 95% IC were used to present the results depending on the data distribution, which was evaluated with Shapiro-Wilk test. Comparison of variables between two groups were performed using the t-student test or Mann-Whitney U test depending on Shapiro-Wilk test results. Comparisons of variables between three groups were performed with Kruskal-Wallis test and pairwise comparison of subgroups according to Conover was used for post-hoc testing. Correlations between variables were estimated with Spearman test. The diagnostic accuracy of single biomarkers was evaluated using logistic regression. For the selected markers, the receiver operating characteristic (ROC) curves were obtained and the area under curve (AUC) was calculated with 95% confidence intervals according to the nonparametric method. ROC curves AUCs were compared using the method of Delong et al. [26] with 2-sided tests at unadjusted α = 0.05 significance levels. The cut point on each marker panel’s ROC curve was established that maximized its discriminative index (Youden index), and the panel’s sensitivity and specificity at that cut point was obtained. A *p*-value less than 0.05 was considered statistically significant. All tests were two-sided. Data were analyzed with MedCalc (MedCalc Software) version 12.2.1.

## Results

### Comparisons of marker concentration in EC, endometriosis and control samples

**Fig. 1:**
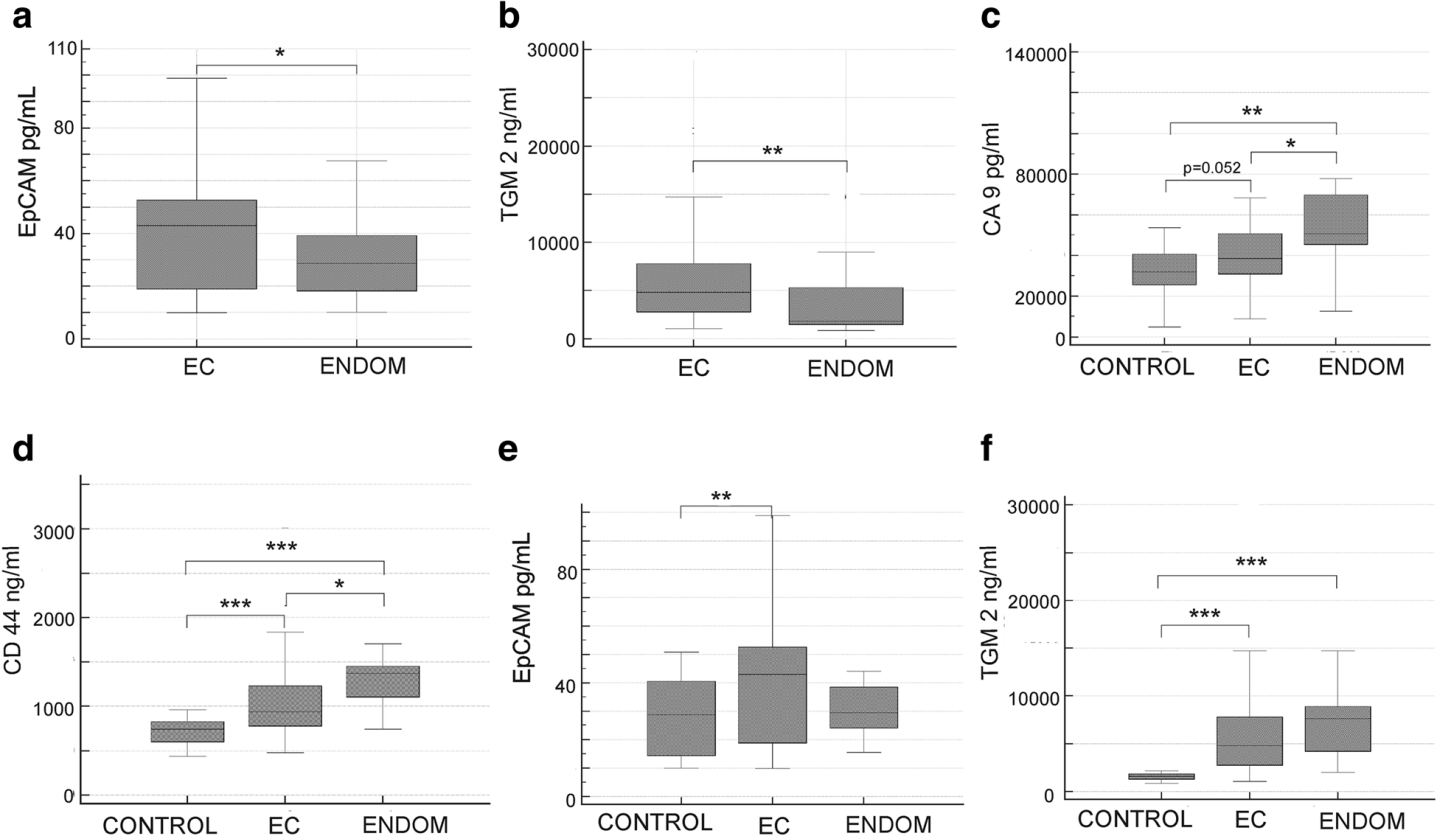
Concentrations of biomarkers in plasma. **a** EpCAM concentration in EC vs. non-EC samples: *median* 44.99 (95% CI 34.80–49.08) vs. 28.67 (95% CI 23.93–35.79), (*U* = 489.50, *Z* = 2.2, *p* = 0.028). **b** TGM2 concentration in EC vs. non-EC samples: *median* 4808.36 (95% CI 3688.71–6224.69) vs. 1828.96 (95% CI 1574.94–3028.34), (*U* = 316, *Z* = 2.85, *p* = 0.004). **c** CA9 concentration in EC vs. control vs. endometriosis (ENDOM) samples. **d** CD44 concentration in EC vs. control vs. endometriosis samples. **e** EpCAM concentration in EC vs. control vs. endometriosis samples. **f** TGM2 concentration in EC vs. control samples vs. endometriosis samples. * *p* < 0.05, ** *p* < 0.01, *** *p* < 0.001

Concentrations of EpCAM (*p* = 0.028) and TGM2 (*p* = 0.004), were significantly increased in plasma of patients with EC as compared to non-EC samples (Fig. 1a, b). There was a significantly increased concentration of midkine in non-EC samples (*p* = 0.035), but the subsequent subgroup analysis revealed that it was due to highly increased midkine level in endometriosis samples (S2-S3).

As compared to healthy controls EC samples were characterized by significantly increased concentrations of CD44 (*p* < 0.001), EpCAM (*p* = 0.033) and TGM2 (*p* < 0.001) (Fig. 1d-f). Concentration of CA9 was also higher in EC samples and the difference approached statistical significance (*p* = 0.052) (Fig. 1c).

Seven analytes differentiated endometriosis and control samples. Six analytes were increased in patients with endometriosis: ALDH1A (*p* = 0.002), CA9 (*p* = 0.005), CD44 (*p* < 0.001), hepsin (*p* = 0.008), midkine (*p* < 0.001), and TGM2 (*p* < 0.001), whereas concentration of a single marker – kallikrein-6, was found to be decreased (*p* = 0.006) (S2, S3).

**Fig. 2:**
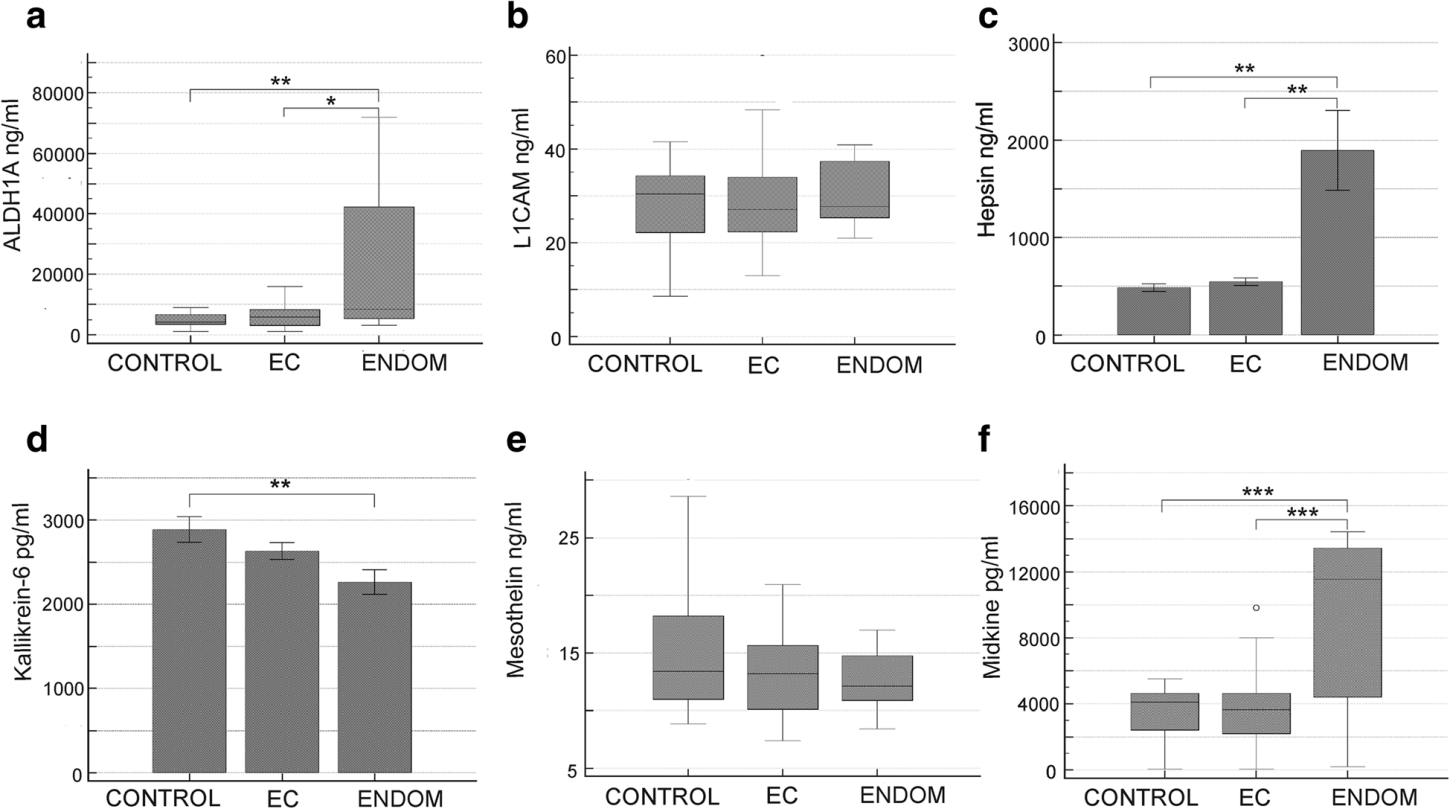
Comparison of biomarkers’ concentrations in endometrial cancer (EC), control, and endometriosis (ENDOM) plasma samples. **a** ALDH1A1. **b** L1CAM. **c** Hepsin. **d** Kallikrein-6. **e** Mesothelin. **f** Midkine. * *p* < 0.05, ** *p* < 0.01, *** *p* < 0.001

Significant differences were encountered between endometriosis and EC in case of five analytes, of which all were increased: ALDH1A (*p* = 0.019), CA9 (*p* = 0.031), CD44 (*p* = 0.010), hepsin (*p* = 0.007), midkine (*p* < 0.001) (Figs. 1, 2).

Descriptive statistics of markers’ concentrations and inferential tests’ results for all comparisons were provided in the Additional file 2: Supplementary Table 1 and Additional file 3: Supplementary Table 2. Additional file 4: Supplementary Table 3, Additional file 5: Supplementary Table 4, Additional file 6: Supplementary Table 5 present correlation analysis results of analytes’ concentrations in EC, endometriosis and control subgroups .

### Concentrations of studied analytes and clinicopathological features of endometrial cancer

For the purpose of the analysis EC patients were divided into three subgroups based on FIGO classification: FIGO IA, FIGO IB and FIGO III. Patients with higher stage were combined into one group due to small numbers of those patients. The group consisting of patients presenting with FIGO IA stage and histological grade 1 was also distinguished (FIGO IA-G1), which represented the earliest phase of disease progression.

**Fig. 3:**
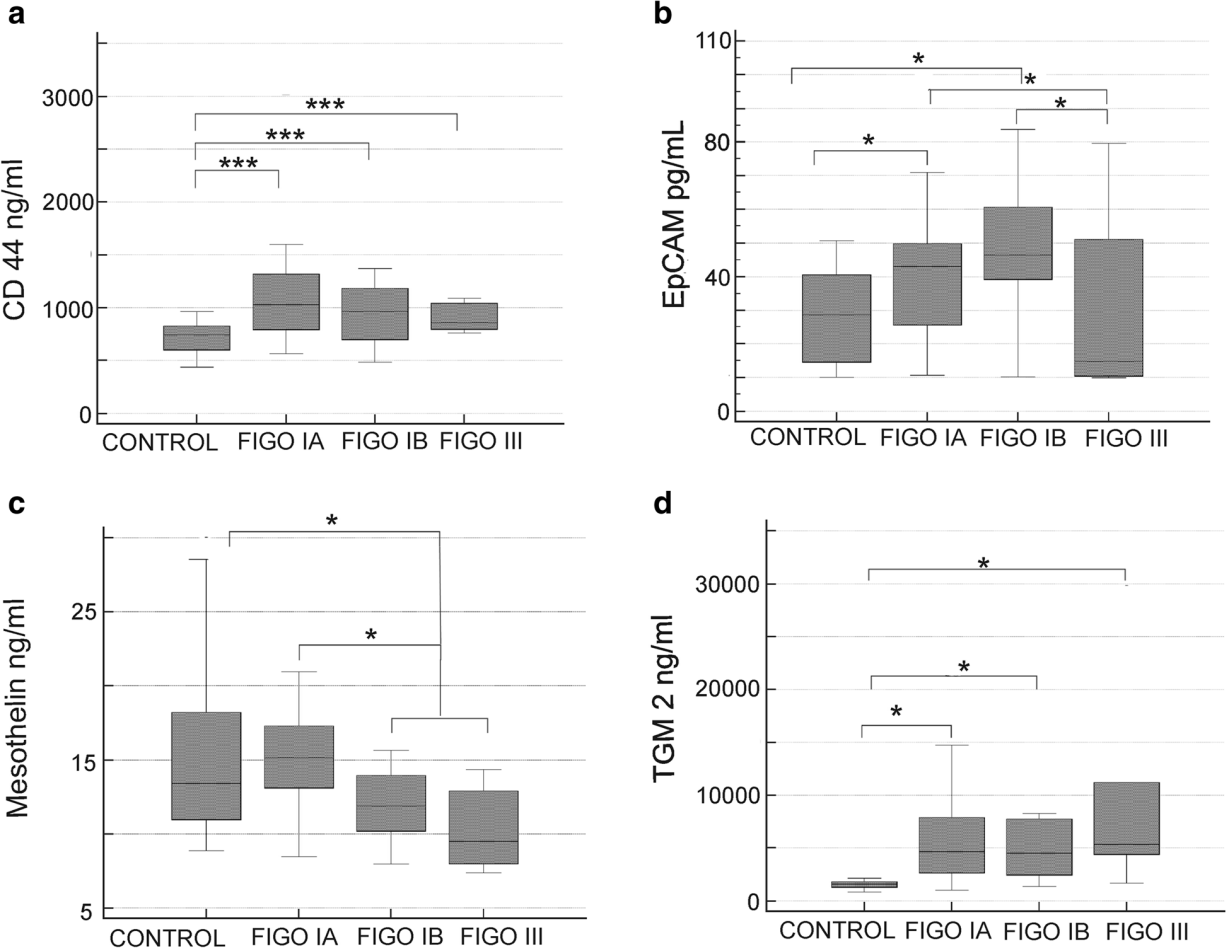
Concentrations of biomarkers in EC plasma samples depending on FIGO stage. **a** CD44 (t(3) = 16.10, *p* = 0.001, post-hoc *p* < 0.05). **b** EpCAM (*t*(3) = 12.06, *p* = 0.007, post-hoc *p* < 0.05). **c** Mesothelin (*t*(2) = 6.15, *p* = 0.046, post-hoc *p* < 0.05). **d** TGM2 (t(3) = 26.04, *p* < 0.001, post-hoc *p* < 0.05). * *p* < 0.05, ** *p* < 0.01, *** *p* < 0.001

Significant differences were encountered in CD 44 and TGM2 concentrations between control samples and each of the FIGO stage (Fig. 3a, d). The two markers were also increased in FIGO IA-G1 group (CD44: *p* < 0.001, post-hoc *p* < 0.05, and TGM2: *p* < 0.001, post-hoc *p* < 0.05). No significant differences were however encountered between individual FIGO stages in regards to CD44 and TGM2 concentrations.

EpCAM concentration was higher in FIGO IA and IB stages as compared to FIGO III group (Fig. 3b). Median EpCAM concentration was low in FIGO III group, however as it was accompanied by large 95% CI (10.00 and 67.60 pg/mL), which could reflect clinical heterogeneity of that group, it was not significantly different from control samples. It was also found that EpCAM concentration in FIGO IA (*p* = 0.025), and in FIGO1B samples was significantly higher comparing to controls (*p* = 0.003).

Significantly higher mesothelin concentration was found in FIGO IA as compared to FIGO IB - III samples (*p* = 0.011), and between FIGO I and FIGO III samples (*p* = 0.038). It was also found that concentration of mesothelin in FIGO IB – III samples was significantly lower comparing to control samples (*p* = 0.018) (Fig. 3c).

Multiple correlations were found between analytes concentrations in EC and endometriosis groups, whereas only one pair of markers (kallikrein-6/midkine) were correlated in control group. No correlations were found between studied analytes and patients’ age (S4-S6).

The associations between altered levels of circulating CD44, TGM2 and EpCAM and hematologic prognostic parameters reflecting a systemic immune and inflammatory response, such as neutrophil/lymphocyte ratio (NLR), monocyte count, and platelet/lymphocyte ratio (PLR) were also evaluated in patients with endometriosis and with endometrial cancer and are presented in the Additional file 7: Table S6 (S7). No significant correlations were found for endometrial cancer and control cases, whereas in endometriosis group monocytes count correlated strongly with CD44 and EpCAM levels, and NLR was significantly correlated with concentration of CD44.

### Logistic regression analysis

#### Regression models for discrimination EC and non-EC samples

**Fig. 4:**
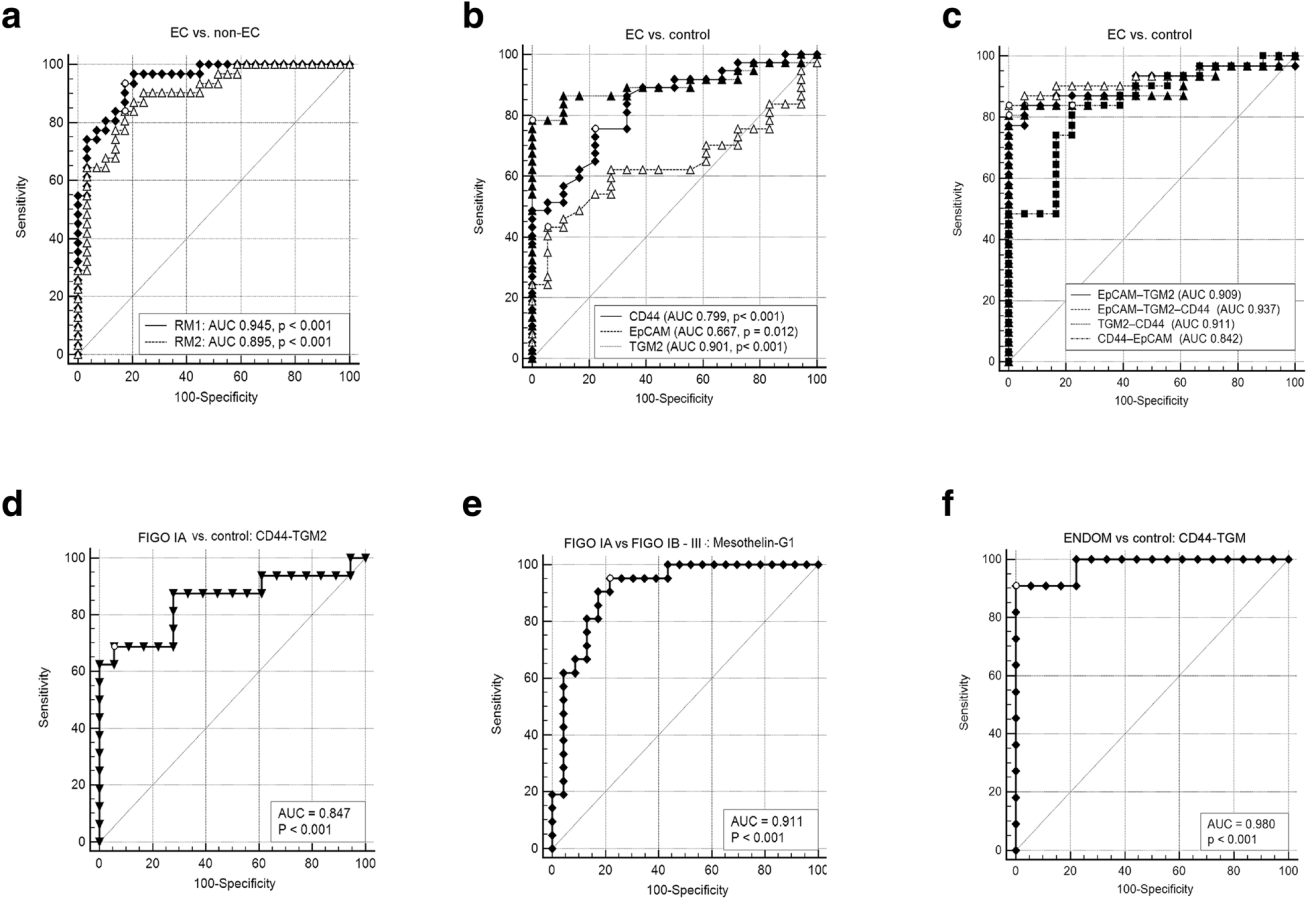
Receiver operating characteristic (ROC) curves. **a** ROC curves for regression models discriminating EC from non-EC samples; RM1 (♦ - 8-marker model: ALDH1A1, CA9, CD44, Hepsin, Kallikrein 6, L1CAM, Midkine, TGM2), RM2 (△ - 5-marker model: ALDH1A1, CD44, EpCAM, Midkine, TGM2). **b** ROC curves for single makers discriminating EC from control samples (▲ - TGM2; ♦ - CD44; △ - EpCAM). **c** ROC curves for regression models discriminating EC from control samples, *p*-value for all AUCs was < 0.001 (♦ - EpCAM-TGM2; △ - EpCAM-TGM2-CD44; ▲ - TGM2-CD44; ■ - CD44-EpCAM). **d** ROC curve for regression model discriminating EC FIGO IA samples from control samples (▼ - CD44-TGM2). **e** ROC curve for regression model discriminating EC FIGO IA samples from FIGO IB - III samples (♦ - Mesothelin-G1). **f** ROC curve for regression model discriminating endometriosis samples from control samples (♦ - CD44-TGM2)

Regression analysis retrieved two models capable of discrimination of EC and non-EC samples with AUC of 0.895 (RM2: 5-marker model, *SE* 0.04, 95% CI 0.79–0.96, *p* < 0.001) and 0.945 (RM1: 8-marker model, *SE* 0.23, 95% CI 0.86–0.99, *p* < 0.001) (Fig. 4a, Table 3).

**Table 3:** Receiver operating characteristic (ROC) curves results obtained for selected biomarkers and logistic regression models

Model	Sensitivity (%)	Specificity (%)	ROC AUC	95% CI	PPV	NPV	Difference in AUC	*p*-value
EC vs. non-EC								
8-marker	93	83	0.945	0.86–0.99	–	–	–	–
5-marker	84	83	0.895	0.79–0.96	–	–	0.05	0.11
EC vs. control*								
CD44/EpCAM/TGM2	84	100	0.937	0.84–0.96	100	99.9	–	–
CD44/EpCAM	82	75	0.842	0.73–0.92	0.85	99.9	0.09	0.067
CD44/TGM2	81	100	0.911	0.8–0.97	100	99.9	0.03	0.209
EpCAM/TGM2	84	94	0.909	0.79–0.97	3.52	99.9	0.02	0.212
CD44	49	100	0.834	0.71–0.92	100	99.8	0.11	0.027
EpCAM	42	95	0.667	0.54–0.78	2.14	99.8	0.3	< 0.001
TGM2	78	100	0.901	0.79–0.97	100	99.9	0.04	0.206
FIGO1A vs. control								
CD44/TGM2	69	94	0.847	0.68–0.95	2.9	99.9	–	–
FIGO1A vs. FIGO1B-4								
Mesothelin/G1	95	78	0.911	0.79–0.98	–	–	–	–
Endometriosis vs. control								
CD44/TGM2	91	100	0.98	0.85–1.00	100	99.1	–	–
EC vs. endometriosis								
ALDH1A1/Midkine	100	73	0.897	0.76–0.97	–	–	–	–

*prevalence of EC (0.26%) used for calculation of PPV and NPV was based on data from https://gis.cdc.gov/Cancer/USCS/DataViz.html

8-marker model: ALDH1A1, CA9, CD44, Hepsin, Kallikrein 6, L1CAM, Midkine, TGM2

5-marker model: ALDH1A1, CD44, EpCAM, Midkine, TGM2

#### Regression models for discrimination EC and control samples

In order to evaluate the diagnostic value of the analytes, which were significantly different in EC samples, the ROC curves were plotted and the areas under the ROC curves (AUC) were calculated and compared. The highest AUC value was calculated for TGM2 and that result was significantly higher in comparison to CD44 and EpCAM (Table 3, Fig. 4b).

Multivariable logistic regression models were then constructed to evaluate, if the diagnostic accuracy would increase with the input of two or three (CD44, TGM2 and EpCAM) biomarkers. The logistic regression *backward* method retrieved model consisting of EpCAM (*b* = 0.04, *SE* 0.02, *p* = 0.08, OR 1.04) and TGM2 (*b* = 0.002, *SE* 0.0007, *p* = 0.008, OR 1.00) as most suitable for discrimination between EC and control samples. Subsequently, models were created using logistic regression *enter* method. Out of the four models obtained in that analysis, the 3-marker model was characterized by the highest AUC of 0.937 (84% sensitivity, 100% specificity). Comparison of the AUCs revealed no significant difference between CD44/TGM2/EpCAM model, the TGM2 and all three 2-marker models (Table 3, Fig. 4c). In an attempt to discriminate early stage EC samples (FIGO IA) from the controls a separate analysis was conducted and retrieved a model consisting of TGM2 (*b* = 0.0006, *SE* 0.0002, *p* = 0.023) and CD44 (*b =* 0.00003, *SE* 0.00002, *p* = 0.034), which distinguished FIGO IA samples from the controls with the 69% sensitivity and 94% specificity and AUC of 0.847 (*SE* 0.07, 95% CI 0.68–0.95, *p* < 0.001) (Fig. 4d).

Another logistic regression analysis was performed to develop strategy for discrimination between FIGO IA samples and more advanced stages of EC. That calculation included grading as one of the variables and retrieved model consisting of mesothelin (*b* = − 0.57, *SE* 0.21, *p* = 0.006, OR 0.57) and histological grade 1 (*b* = − 2.86, *SE* 1.05, *p* = 0.007, OR 0.06), as factors predicting the lower progression of EC. The ROC curve plotted based on that model retrieved AUC of 0.911 (*SE* 0.05, 95% CI 0.79–0.98, *p* < 0.001), 95% sensitivity, and 78% specificity (Fig. 4e).

It was also found that a regression model consisting of CD44 and TGM2 discriminated endometriosis from control samples with high accuracy (Fig. 4f, Table 3, AUC 0.98, 95% CI 0.85–1.00, *p* < 0.001), however this result needs to be considered as preliminary due to the small number of endometriosis samples.

## Discussion

Several plasma or serum markers were proposed in endometrial cancer diagnosis, however none has attained the high accuracy predisposing to the rank of a screening tool [4]. Despite new developments in cancer management a gradual increase of 1.6% a year in endometrial carcinoma death rates was noted over 2006–2015 [2, 3]. Beyond any doubt, early detection of endometrial cancer could increase survival rates and decrease morbidity connected to this disease.

Majority of the analytes chosen for the presented study has not been studied previously in the blood derived samples of endometrial cancer patients, however their roles in EC pathogenesis were suggested by immunohistochemistry (ICH) or gene expression studies [5–25]. The multiplex ELISA-based approach was undertaken by our team, as it allows for simultaneous detection of several analytes in one sample. Such method reduces technical errors and increases accuracy. It can be also easily translated into clinical practice.

Accurate tool in endometrial cancer diagnosis needs to be sensitive but also specific, as this carcinoma rarely occurs as a lone disorder. It is often associated with obesity, hypertension and diabetes, but can be also accompanied by endometriosis [27]. In line with that assumption endometriosis samples were included in our study.

Analysis of data revealed that concentrations of CD44, EpCAM, and TGM2 were significantly increased in EC comparing to control samples. It was also found that concentrations of the two stem-cell markers, CD44 and TGM2 were significantly correlated in EC samples. These results correspond to results of increased CD44 expression in EC tissues reported by Elbasateeny et al. and Wojciechowski et al., as well as upregulation of EpCAM in serous uterine cancers presented by El-Sahwi et al. [8, 9, 11]. TGM2 has been previously indicated in the pathogenesis of a number of cancers including ovarian cancer, however our finding of its highly increased concentration in EC plasma samples seems to be a new discovery [25]. Concentration of CA9 was also increased in EC samples as compared to controls and the difference was very close to statistical significance. That result was in line with ICH studies performed by other authors [6, 7]. Kalikrein-6 levels were investigated in tissues and plasma samples of endometrioid adenocarcinoma by Santin et al., and similarly to our results, the authors did not find differences between cancer and healthy patients [15]. Kalikrein-6 levels were, however, highly increased in uterine serous papillary cancer, in both tissues and plasma samples [16]. L1CAM has been suggested as a strong prognostic marker by several ICH studies [17–20]. Unfortunately, we did not reproduce those results. We speculate, that it could be due to relatively small number of advanced EC cases in our study population. Our results could be also justified by the observation of Pasanen et al., who observed no difference between L1CAM serum levels of patients with an L1CAM-positive or L1CAM-negative endometrial carcinoma [18].

High tissue expression of mesothelin has been linked to aggressive tumor behavior and worse prognosis in ovarian and pancreatic cancers as well as in mesothelioma [28]. The diagnostic value of circulating mesothelin has been also suggested in those neoplasms [29, 30]. In our study, mesothelin plasma concentrations did not differ between EC and healthy samples. However, comparison of controls to more advanced cases (FIGO IB-III) revealed significantly lower concentration of that protein in plasma from EC patients. Moreover, mesothelin concentration in FIGO IB-III samples was decreased as compared to FIGO IA stage. There was a marked trend towards decreased mesothelin concentration with the progression of the disease. Few studies on mesothelin expression in EC tissues revealed a moderate expression level and to date there no available studies investigating soluble mesothelin levels in EC [21, 22, 28]. Therefore, the somewhat surprising results obtained in this study require further investigations combining tissue and blood derived samples from the same patients. The soluble form of mesothelin is likely due to an abnormal splicing event, but it is also possible, that it is a proteolytically cleaved fragment of membrane-bound mesothelin [31]. It has been recently revealed that mesothelin binds to Ca 125 and that this interaction mediates cell adhesion [32]. Therefore, one of the possible hypotheses behind the phenomenon of lower circulating mesothelin levels in advanced EC, could be connected to decreased release of the glycoprotein from the tumor cells due its enhanced utilization within the tumor microenvironment, including bounding to Ca 125, which expression is also increased in more aggressive ECs.

When concentrations of other analytes were compared according to clinicopathological characteristics the most important finding regarded EpCAM, which concentration was higher in FIGO IA as compared to more advanced stages (>FIGO IA). No differences in concentrations of other markers were found in regards to histological grading, myometrial invasion and FIGO staging. The lack of correlation of CD44 level with clinicopathological features despite its highly significant increase in EC samples corresponds to the results obtained by other authors [7, 8].

Further analyses including the subset of endometriosis samples revealed interesting findings. Concentration of seven analytes (ALDH1A, CA9, CD44, hepsin, midkine, TGM2, and kallikrein-6) differed in endometriosis samples as compared to control samples and levels of five markers (ALDH1A, CA9, CD44, hepsin, and midkine) were different between endometriosis and EC samples. We acknowledge that those results can only be regarded as preliminary findings, due to a small number of patients with endometriosis. However, they seem to explain the observation, that only three analytes were different (EpCAM, midkine and TGM2) in EC in comparison to non-EC group.

Regression analysis revealed panels of analytes characterized by high diagnostic accuracy in discriminating EC samples. Firstly, TGM2 was found to be a highly accurate, single marker able to differentiate between EC and healthy controls with the AUC of 0.901 (78% sensitivity, 100% specificity). Secondly, multivariable logistic regression models were constructed to evaluate, if the input of several biomarkers would improve the diagnostic accuracy. The *backward* analysis method retrieved the model consisting of EpCAM and TGM2 as most suitable model for discrimination between EC and controls, which yielded AUC of 0.909 with 84% sensitivity and 94% specificity. Subsequently, regression models were created using the *enter* method and different combinations of CD44, TGM2, and EpCAM. Out of the four models obtained in that analysis, the 3-marker model was characterized by the highest AUC of 0.937 with sensitivity of 84 and 100% specificity. Comparison of ROCs revealed that the AUC for CD44/TGM2/EpCAM model did not differ significantly from the AUC of TGM2 and all three 2-marker models. Analysis aiming at discrimination between EC samples and non-EC group, which included endometriosis samples, required input of least five markers to obtain a satisfactory AUC of 0.895, and the utilization of eight markers was necessary to increase the AUC to 0.945.

Comparing to the work of other authors, marker panels discovered in this study seem to offer similar or better accuracy. Yurkovetsky et al. reported prolactin as the strongest single biomarker for EC with 98.3% sensitivity and 98.0% specificity and the 5-marker panel (prolactin, GH, Eotaxin, E-selectin, and TSH), which yielded high sensitivity and specificity in discrimination between EC, ovarian and breast cancers [33]. Another study suggested that the panel of ApoA-I, TTR, and TF distinguished normal samples from early-stage endometrial cancer with a sensitivity of 71% (specificity, 88%) and normal samples from late stage endometrial cancer with a sensitivity of 82% [34]. CA 125 and HE4 were also extensively studied as single biomarkers or in combination and yielded no more than low to moderate accuracy depending on the study [35–39]. Although few studies have proven acceptable accuracy of CA 125/HE4 panel, it needs to be acknowledged, that both CA-125 and HE4 can be elevated in various malignancies and benign pathologies of reproductive tract [35]. Therefore, their specificity for endometrial tumors is questionable. What is more, the distinct effects of physiological factors on prolactin secretion shadow the credibility of this hormone in early diagnosis of endometrial tumors [40].

EC has a very good prognosis providing it is diagnosed at the early stage of clinical progression. Nowadays most EC are discovered only after the clinical signs are already present, and the screening method is still not available [2]. One of the research questions of the presented study was to verify, if one of the combinations of studied markers would be able to discern those early cases (FIGO IA) from healthy population. For that purpose, logistic regression analysis was conducted and retrieved a model consisting of TGM2 and CD44 with the 69% sensitivity and 94% specificity and AUC of 0.847. This finding is in line with the study of Elbasateeny et al., who reported that CD44 along with CD133 might participate in early-stage endometrial cancer carcinogenesis, and their overexpression might facilitate early diagnosis of endometrial cancers [9].

Accurate preoperative diagnosis of the disease progression has an important clinical significance in endometrial cancer management, as early EC does not require lymphadenectomy and radical surgery. A method with reasonable accuracy in depicting tumors at the earliest stage of progression could decrease morbidity and complications connected with unnecessary lymphadenectomies and extensive resection of pelvic tissues. In line with that notion a multivariable regression analysis was performed to discriminate between FIGO IA samples and more advanced stages of EC. That analysis incorporated histological grading as one of the variables and retrieved model consisting of mesothelin concentration and histological grade 1, as factors predicting early clinical stage of EC. The ROC curve plotted based on that model retrieved AUC of 0.911, with 95% sensitivity and 78% specificity.

These results present similar accuracy as compared to blood derived markers reported by other research groups [30, 35, 41].

There are some limitations to our study that need to be addressed. Firstly, although we investigated multiple markers the number of patients included in the study was only moderate. Therefore, we consider obtained results as preliminary, which need to be repeated in larger population, preferably in the multicenter setting. Secondly, we acknowledge that the research would benefit from tissue expression analysis. We plan to address these issues in the future studies.

## Conclusions

In conclusion, in this study we report novel, potential biomarkers that could offer a good diagnostic accuracy in diagnosis of EC, with TGM2 being reported for the first time as plasma marker. In addition, we point to the fact, that endometriosis might share similarities in the pattern of markers alterations characteristic for EC, what leads to the conclusion that its undiagnosed presence could alter results of EC studies and need to be taken into consideration during the research design.

## Additional files

Additional file 1: The dataset: clinicopathological characteristics of the endometrial cancer patients. (XLSX 10 kb)

Additional file 2: **Table S1.** Descriptive statistics of the studied analytes’ plasma concentrations. (DOCX 19 kb)

Additional file 3: **Table S2.** Statistical tests’ results for the comparisons between groups: Mann-Whitney (M-W) or t-test were used based on Shapiro-Wilk test results. (DOCX 19 kb)

Additional file 4: **Table S3.** Results of the correlation analysis for the analytes in the EC group. (DOCX 26 kb)

Additional file 5: **Table S4.** Results of the correlation analysis for the analytes in the control group. (DOCX 27 kb)

Additional file 6: **Table S5.** Results of the correlation analysis for the analytes in the endometriosis group. (DOCX 27 kb)

Additional file 7: **Table S6.** Correlation analysis results of CD44, TGM2 and EpCAM plasma levels and neutrophil/lymphocyte ratio (NLR), monocyte count, and platelet/lymphocyte ratio (PLR). (DOCX 14 kb)

Additional file 8: The dataset supporting the conclusions of this article. (XLSX 17 kb)

## Abbreviations

ALDH1A1: Aldehyde dehydrogenase 1 Family, Member A1,
AUC: Area under curve
CA9: Carbonic Anhydrase anhydrase IX
EC: Endometrial cancer
EDTA: Ethylenediaminetetraacetic acid
ELISA: EnzymeEnzyme-linked immunosorbent assay
EpCAM: Epithelial cell adhesion molecule
FIGO: International federation of gynecology and obstetrics
ICH: IImmunohistochemistry
L1CAM: Neural cell adhesion Molecule L1
NLR: Neutrophil/lymphocyte ratio
PLR: Platelet/lymphocyte ratio
ROC: Curve receiver operating characteristic curve
TGM2: Transglutaminase 2
