# Supplementary material for: CD44, TGM2 and EpCAM as novel plasma markers in endometrial cancer diagnosis
Source: BMC Cancer. 2019 Apr 29;19:401. doi: 10.1186/s12885-019-5556-x (PMC6489287; doi:10.1186/s12885-019-5556-x)
Supplement: Supplementary file 2 — Table S1. Descriptive statistics of the studied analytes’ plasma concentrations. (DOCX 19 kb) [file 12885_2019_5556_MOESM2_ESM.docx]

Table S1. Descriptive statistics of the studied analytes.

| Analyte | EC  *n* = 45 | | non-EC  *n* = 31 | | Control  *n* = 20 | | Endometriosis  *n* = 11 | |
| --- | --- | --- | --- | --- | --- | --- | --- | --- |
|  | *Median* | *95% CI* | *Median* | *95% CI* | *Median* | *95% CI* | *Median* | *95% CI* |
| ALDH1A1 | 5879.75 | 4760.74-7425.62 | 4820.52 | 4083.36-7401.39 | 4219.93 | 3688.90-5928.17 | 8526.45 | 4775.49-48808.89 |
| CA9 | 38128.8 | 32886.6-42944.1 | 37382.54 | 29939.66-46012.1 | 31864.76 | 26189.4-39326.7 | 50579.77 | 42730.9-72304.1 |
| CD44 | 949.61 | 851.69-1066.66 | 798.94.3 | 739.88-959.59 | 739.49 | 618.46-798.94 | 1371.56 | 1025.35-1498.42 |
| EpCAM | 44.99 | 34.80-49.08 | 28.67 | 23.93-35.79 | 28.67 | 15.36-38.41 | 29.43 | 23.99-40.19 |
| Hepsin | 487.02 | 400.49-584.34 | 507.85 | 455.55-667.33 | 472.65 | 437.19-541.01 | 2636.31 | 447.21-2636.31 |
| Kallikrein-6 | 2604.68 | 2322.87-2840.50 | 2583.45 | 2198.87-2906.51 | 2768.55 | 2439.17-3174.03 | 2185.50 | 1853.70-2839.24 |
| L1CAM | 27.47 | 24.35-29.18 | 28.51 | 25.82-32.95 | 30.43 | 24.28-33.86 | 27.74 | 24.98-38.08 |
| Mesothelin | 13.47 | 11.83-14.69 | 13.22 | 12.07-15.54 | 13.41 | 12.09-17.81 | 12.09 | 10.62-15.21 |
| Midkine | 3597.62 | 2765.81-3880.53 | 4460.9 | 3875.65-4887.89 | 4108.69 | 3058.95-4563.25 | 11714.24 | 4446.38-13430.02 |
| TGM2 | 4808.36 | 3688.71-6224.69 | 1828.96 | 1574.94-3028.34 | 1574.94 | 1352.06-1815.28 | 7600.00 | 3883.46-10029.44 |
|  | EC  *n* = 45 | | non-EC  *n* = 31 | | Control  *n* = 20 | | Endom  *n* = 11 | |
| Analyte | *Mean* | *95% CI* | *Mean* | *95% CI* | *Mean* | *95% CI* | *Mean* | *95% CI* |
| Hepsin | 538.76 | 464.00-613.52 | - | - | 482.14 | 401.72-562.56 | 1893.58 | 976.97-2810.19 |
| Kallikrein-6 | 2604.99 | 2401.79-2808.2 | - | - | 2887.39 | 2571.12-3203.66 | 2262.63 | 1936.34-2588.92 |
