# Supplementary material for: CD44, TGM2 and EpCAM as novel plasma markers in endometrial cancer diagnosis
Source: BMC Cancer. 2019 Apr 29;19:401. doi: 10.1186/s12885-019-5556-x (PMC6489287; doi:10.1186/s12885-019-5556-x)
Supplement: Supplementary file 3 — Table S2. Statistical tests’ results for the comparisons between groups: Mann-Whitney (M-W) or t-test were used based on Shapiro-Wilk test results. (DOCX 19 kb) [file 12885_2019_5556_MOESM3_ESM.docx]

Table S2. Statistical tests results for the comparisons between groups: Mann-Whitney (M-W) or t-test were used based on Shapiro-Wilk test results.

| Analyte | EC vs. non-EC | | | EC vs. control | | | Endometriosis vs. control | | | EC vs. endometriosis | | |
| --- | --- | --- | --- | --- | --- | --- | --- | --- | --- | --- | --- | --- |
| M-W test | *U* | *Z* | *p* | *U* | *Z* | *p* | *U* | *Z* | *p* | *U* | *Z* | *p* |
| ALDH1A1 | 645.00 | 0.07 | 0.95 | 319.00 | 1.52 | 0.13 | 36.00 | 3.06 | 0.002 | 124.00 | 2.35 | 0.019 |
| CA9 | 665.00 | 0.34 | 0.74 | 313.5 | 1.94 | 0.052 | 41.5 | 2.83 | 0.005 | 143.00 | 2.16 | 0.031 |
| CD44 | 552.5 | 1.53 | 0.13 | 180.5 | 3.83 | 0.0001 | 9.5 | 4.15 | <0.0001 | 123.00 | 2.57 | 0.01 |
| EpCAM | 489.5 | 2.2 | 0.028 | 300.00 | 2.13 | 0.033 | 92.00 | 0.74 | 0.46 | 189.5 | 1.2 | 0.23 |
| Hepsin | 584.00 | 1.06 | 0.29 | - | - | - | - | - | - | - | - | - |
| Kallikrein-6 | 664.00 | 0.35 | 0.72 | - | - | - | - | - | - | - | - | - |
| L1CAM | 605.00 | 0.67 | 0.5 | 405.00 | 0.36 | 0.72 | 96.5 | 0.56 | 0.58 | 199.00 | 0.79 | 0.43 |
| Mesothelin | 634.5 | 0.51 | 0.61 | 385.5 | 0.79 | 0.43 | 89.00 | 0.87 | 0.39 | 235.00 | 0.15 | 0.88 |
| Midkine | 462.00 | 2.11 | 0.035 | 387.5 | 0.49 | 0.62 | 24.00 | 3.34 | 0.0008 | 35.5 | 4.05 | 0.0001 |
| TGM2 | 316.00 | 2.85 | 0.004 | 66.00 | 4.79 | <0.0001 | 4.00 | 4.27 | <0.0001 | 157.00 | 1.14 | 0.25 |
| t-test | *d(f)* | *t(d)* | *p* | *d(f)* | *t(d)* | *p* | *d(f)* | *t(d)* | *p* | *d(f)* | *t(d)* | *p* |
| Hepsin |  |  |  | 62 | -0.93 | 0.29 | 10.2 | 3.28 | 0.008 | 10.2 | 3.42 | 0.007 |
| Kallikrein-6 | - | - | - | 36.6 | 1.56 | 0.13 | 26.7 | -2.97 | 0.006 | 54 | -1.56 | 0.12 |
