# Supplementary material for: CD44, TGM2 and EpCAM as novel plasma markers in endometrial cancer diagnosis
Source: BMC Cancer. 2019 Apr 29;19:401. doi: 10.1186/s12885-019-5556-x (PMC6489287; doi:10.1186/s12885-019-5556-x)
Supplement: Supplementary file 4 — Table S3. Results of the correlation analysis for the analytes in the EC group. (DOCX 26 kb) [file 12885_2019_5556_MOESM4_ESM.docx]

Table S3. Results of correlation analysis for the analytes in the EC group.

| Correlation table | | ALDH1A1 | CA9 | CD44 | EpCAM | Hepsin | Kallikrein 6 | L1CAM | Mesothelin | Midkine | TGM2 |
| --- | --- | --- | --- | --- | --- | --- | --- | --- | --- | --- | --- |
| ALDH1A1 | *R*  *p* |  | 0.020 0.8990 | 0.078 0.6228 | -0.082 0.6063 | -0.164 0.3008 | 0.170 0.2811 | 0.448 0.0038 | 0.161 0.3142 | -0.079 0.6332 | -0.012 0.9446 |
| CA9 | *R*  *p* | 0.020 0.8990 |  | 0.213 0.1605 | 0.450 0.0019 | 0.565 0.0001 | 0.288 0.0553 | 0.243 0.1161 | 0.252 0.0986 | 0.500 0.0007 | 0.194 0.2496 |
| CD44 | *R*  *p* | 0.078 0.6228 | 0.213 0.1605 |  | -0.057 0.7077 | 0.254 0.0965 | 0.119 0.4365 | 0.079 0.6157 | 0.127 0.4097 | 0.357 0.0202 | 0.443 0.0060 |
| EpCAM | *R*  *p* | -0.082 0.6063 | 0.450 0.0019 | -0.057 0.7077 |  | 0.273 0.0732 | 0.013 0.9319 | 0.327 0.0322 | -0.038 0.8083 | 0.286 0.0659 | -0.038 0.8250 |
| Hepsin | *R*  *p* | -0.164 0.3008 | 0.565 0.0001 | 0.254 0.0965 | 0.273 0.0732 |  | -0.148 0.3371 | 0.192 0.2229 | -0.024 0.8796 | 0.370 0.0174 | 0.081 0.6367 |
| Kallikrein-6 | *R*  *p* | 0.170 0.2811 | 0.288 0.0553 | 0.119 0.4365 | 0.013 0.9319 | -0.148 0.3371 |  | 0.219 0.1586 | 0.377 0.0116 | -0.040 0.8032 | 0.018 0.9167 |
| L1CAM | *R*  *p* | 0.448 0.0038 | 0.243 0.116 | 0.079 0.6157 | 0.327 0.0322 | 0.192 0.2229 | 0.219 0.1586 |  | 0.224 0.1534 | 0.073 0.6542 | -0.215 0.2144 |
| Mesothelin | *R*  *p* | 0.161 0.3142 | 0.252 0.0986 | 0.127 0.4097 | -0.038 0.8083 | -0.024 0.8796 | 0.377 0.0116 | 0.224 0.1534 |  | 0.096 0.5442 | -0.077 0.6547 |
| Midkine | *R*  *p* | -0.079 0.6332 | 0.500 0.0007 | 0.357 0.0202 | 0.286 0.0659 | 0.370 0.0174 | -0.040 0.8032 | 0.073 0.6542 | 0.096 0.5442 |  | 0.159 0.3601 |
| TGM2 | *R*  *p* | -0.012 0.9446 | 0.194 0.2496 | 0.443 0.0060 | -0.038 0.8250 | 0.081 0.6367 | 0.018 0.9167 | -0.215 0.2144 | -0.077 0.6547 | 0.159 0.3601 |  |
