# Supplementary material for: CD44, TGM2 and EpCAM as novel plasma markers in endometrial cancer diagnosis
Source: BMC Cancer. 2019 Apr 29;19:401. doi: 10.1186/s12885-019-5556-x (PMC6489287; doi:10.1186/s12885-019-5556-x)
Supplement: Supplementary file 5 — Table S4. Results of the correlation analysis for the analytes in the control group. (DOCX 27 kb) [file 12885_2019_5556_MOESM5_ESM.docx]

Table S4. Results of correlation analysis for the analytes in the control group.

| Correlation table | | ALDH1A1 | CA9 | CD44 | EpCAM | Hepsin | Kallikrein 6 | L1CAM | Mesothelin | Midkine | TGM2 |
| --- | --- | --- | --- | --- | --- | --- | --- | --- | --- | --- | --- |
| ALDH1A1 | *R*  *p* |  | 0.062 0.7959 | -0.008 0.9749 | 0.342 0.1397 | -0.072 0.7623 | 0.319 0.1707 | -0.241 0.3051 | 0.394 0.0856 | 0.072 0.7621 | 0.029 0.9093 |
| CA9 | *R*  *p* | 0.062 0.7959 |  | 0.033 0.8896 | 0.263 0.2626 | 0.212 0.3686 | 0.159 0.5033 | 0.349 0.1312 | 0.106 0.6558 | 0.103 0.6670 | -0.349 0.1557 |
| CD44 | *R*  *p* | -0.008 0.9749 | 0.033 0.8896 |  | 0.318 0.1713 | 0.239 0.3096 | -0.032 0.8923 | 0.406 0.0753 | 0.211 0.3709 | -0.130 0.5861 | -0.123 0.6268 |
| EpCAM | *R*  *p* | 0.342 0.1397 | 0.263 0.2626 | 0.318 0.1713 |  | 0.131 0.5823 | 0.120 0.6155 | -0.120 0.6154 | 0.296 0.2045 | 0.140 0.5570 | 0.148 0.5570 |
| Hepsin | *R*  *p* | -0.072 0.7623 | 0.212 0.3686 | 0.239 0.3096 | 0.131 0.5823 |  | 0.138 0.5608 | -0.091 0.7028 | 0.068 0.7768 | -0.303 0.1947 | -0.209 0.4060 |
| Kallikrein-6 | *R*  *p* | 0.319 0.1707 | 0.159 0.5033 | -0.032 0.8923 | 0.120 0.6155 | 0.138 0.5608 |  | 0.031 0.8973 | 0.011 0.9649 | -0.470 0.0363 | -0.323 0.1906 |
| L1CAM | *R*  *p* | -0.241 0.3051 | 0.349 0.1312 | 0.406 0.0753 | -0.120 0.6154 | -0.091 0.7028 | 0.031 0.8973 |  | 0.335 0.1482 | -0.018 0.9410 | -0.259 0.2995 |
| Mesothelin | *R*  *p* | 0.394 0.0856 | 0.106 0.6558 | 0.211 0.3709 | 0.296 0.2045 | 0.068 0.7768 | 0.011 0.9649 | 0.335 0.1482 |  | 0.374 0.1042 | 0.075 0.7662 |
| Midkine | *R*  *p* | 0.072 0.7621 | 0.103 0.6670 | -0.130 0.5861 | 0.140 0.5570 | -0.303 0.1947 | -0.470 0.0363 | -0.018 0.9410 | 0.374 0.1042 |  | 0.095 0.7088 |
| TGM2 | *R*  *p* | 0.029 0.9093 | -0.349 0.1557 | -0.123 0.6268 | 0.148 0.5570 | -0.209 0.4060 | -0.323 0.1906 | -0.259 0.2995 | 0.075 0.7662 | 0.095 0.7088 |  |
