# Supplementary material for: CD44, TGM2 and EpCAM as novel plasma markers in endometrial cancer diagnosis
Source: BMC Cancer. 2019 Apr 29;19:401. doi: 10.1186/s12885-019-5556-x (PMC6489287; doi:10.1186/s12885-019-5556-x)
Supplement: Supplementary file 6 — Table S5. Results of the correlation analysis for the analytes in the endometriosis group. (DOCX 27 kb) [file 12885_2019_5556_MOESM6_ESM.docx]

Table S5. Results of correlation analysis for the analytes in the endometriosis group.

| Correlation table | | ALDH1A1 | CA9 | | CD44 | | EpCAM | | Hepsin | Kallikrein 6 | L1CAM | Mesothelin | Midkine | TGM2 |
| --- | --- | --- | --- | --- | --- | --- | --- | --- | --- | --- | --- | --- | --- | --- |
| ALDH1A1 | *R*  *p* |  | 0.023 0.9469 | 0.182 0.5926 | | 0.200 0.5554 | | 0.076 0.8236 | | 0.127 0.7092 | 0.555 0.0767 | -0.582 0.0604 | -0.395 0.2299 | 0.301 0.3689 |
| CA9 | *R*  *p* | 0.023 0.9469 |  | 0.845 0.0011 | | 0.799 0.0032 | | 0.771 0.0055 | | -0.525 0.0972 | 0.466 0.1488 | 0.411 0.2093 | 0.636 0.0354 | 0.510 0.1088 |
| CD44 | *R*  *p* | 0.182 0.5926 | 0.845 0.0011 |  | | 0.800 0.0031 | | 0.934 <0.001 | | -0.582 0.0604 | 0.636 0.0353 | 0.373 0.2589 | 0.596 0.0528 | 0.569 0.0674 |
| EpCAM | *R*  *p* | 0.200 0.5554 | 0.799 0.0032 | 0.800 0.0031 | |  | | 0.620 0.0420 | | -0.355 0.2847 | 0.318 0.3403 | 0.364 0.2716 | 0.220 0.5153 | 0.196 0.5637 |
| Hepsin | *R*  *p* | 0.076 0.8236 | 0.771 0.0055 | 0.934 <0.001 | | 0.620 0.0420 | |  | | -0.629 0.0380 | 0.553 0.0776 | 0.439 0.1772 | 0.722 0.0122 | 0.621 0.0414 |
| Kallikrein-6 | *R*  *p* | 0.127 0.7092 | -0.525 0.0972 | -0.582 0.0604 | | -0.355 0.2847 | | -0.629 0.0380 | |  | -0.200 0.5554 | -0.118 0.7293 | -0.734 0.0101 | -0.191 0.5730 |
| L1CAM | *R*  *p* | 0.555 0.0767 | 0.466 0.1488 | 0.636 0.0353 | | 0.318 0.3403 | | 0.553 0.0776 | | -0.200 0.5554 |  | -0.282 0.4011 | 0.248 0.4627 | 0.720 0.0125 |
| Mesothelin | *R*  *p* | -0.582 0.0604 | 0.411 0.2093 | 0.373 0.2589 | | 0.364 0.2716 | | 0.439 0.1772 | | -0.118 0.7293 | -0.282 0.4011 |  | 0.413 0.2070 | 0.105 0.7591 |
| Midkine | *R*  *p* | -0.395 0.2299 | 0.636 0.0354 | 0.596 0.0528 | | 0.220 0.5153 | | 0.722 0.0122 | | -0.734 0.0101 | 0.248 0.4627 | 0.413 0.2070 |  | 0.501 0.1163 |
| TGM2 | *R*  *p* | 0.301 0.3689 | 0.510 0.1088 | 0.569 0.0674 | | 0.196 0.5637 | | 0.621 0.0414 | | -0.191 0.5730 | 0.720 0.0125 | 0.105 0.7591 | 0.501 0.1163 |  |
