# Supplementary material for: CD44, TGM2 and EpCAM as novel plasma markers in endometrial cancer diagnosis
Source: BMC Cancer. 2019 Apr 29;19:401. doi: 10.1186/s12885-019-5556-x (PMC6489287; doi:10.1186/s12885-019-5556-x)
Supplement: Supplementary file 7 — Table S6. Correlation analysis results of CD44, TGM2 and EpCAM plasma levels and neutrophil/lymphocyte ratio (NLR), monocyte count, and platelet/lymphocyte ratio (PLR). (DOCX 14 kb) [file 12885_2019_5556_MOESM7_ESM.docx]

Table S6. Correlation analysis results of CD44, TGM2 and EpCAM plasma levels and neutrophil/lymphocyte ratio (NLR), monocyte count, and platelet/lymphocyte ratio (PLR).

|  | | NLR | | | Monocyte count | | | PLR | | |
| --- | --- | --- | --- | --- | --- | --- | --- | --- | --- | --- |
|  |  | Endom | EC | Control | Endom | EC | Control | Endom | EC | Control |
| CD44 | r | 0.82 | 0.2 | -0.07 | 0.735 | -0.05 | 0.068 | -0.367 | -0.036 | 0.221 |
|  | p | 0.002 | 0.19 | 0.75 | 0.01 | 0.73 | 0.77 | 0.26 | 0.81 | 0.34 |
| TGM2 | r | 0.290 | 0.087 | -0.033 | 0.233 | 0.14 | 0.386 | 0.433 | -0.155 | -0.119 |
|  | p | 0.37 | 0.61 | 0.89 | 0.48 | 0.41 | 0.11 | 0.18 | 0.36 | 0.63 |
| EpCAM | r | 0.43 | -0.059 | 0.189 | 0.669 | -0.04 | 0.142 | -0.383 | 0.149 | 0.126 |
|  | p | 0.18 | 0.7 | 0.42 | 0.02 | 0.76 | 0.55 | 0.24 | 0.33 | 0.59 |
| NLR - neutrophil/lymphocyte ratio; PLR - platelet/lymphocyte ratio; Endom – endometriosis; EC – endometrial cancer | | | | | | | | | | |
